# Characteristics of a self-management support programme applicable in primary health care: a qualitative study of users’ and health professionals’ perceptions

**DOI:** 10.1186/s12913-014-0562-9

**Published:** 2014-11-08

**Authors:** Hilde Strøm Solberg, Aslak Steinsbekk, Marit Solbjør, Randi Granbo, Helge Garåsen

**Affiliations:** Department of Public Health and General Practice, Norwegian University of Technology and Science, Post Box 8905, MTFS, N- 7491 Trondheim, Norway; Department of Physiotherapy, Clinic of Clinical Services, St. Olav’s University Hospital, Trondheim, Norway; Department of Social Work and Health Science, Norwegian University of Technology and Science, Trondheim, Norway; Centre of competence for movement disability and falls in older persons, St. Olav’s University Hospital, Trondheim, Norway; Department of Health and Social Services, City of Trondheim, Trondheim, Norway

**Keywords:** Self-care, Patient education as topic, Primary health care, Chronic disease, Health services research, Delivery of health care/Integrated, Qualitative interviews as topic

## Abstract

**Background:**

Development of more self-management support programmes in primary health care has been one option used to enhance positive outcomes in chronic disease management. At present, research results provide no consensus on what would be the best way to develop support programmes into new settings. The aim of the present study was therefore to explore users’ and health professionals’ perceptions of what would be the vital elements in a self - management support programme applicable in primary health care, how to account for them, and why.

**Methods:**

Four qualitative, semi-structured focus group interviews were conducted in Central Norway. The informants possessed experience in development, provision, or participation in a self-management support programme. Data was analysed by the Systematic Text Condensation method.

**Results:**

The results showed an overall positive expectation to the potential benefits of development of a self-management support programme in primary health care. Despite somewhat different arguments and perspectives, the users and the health professionals had a joint agreement on core characteristics; a self-management support programme in primary health care should therefore be generic, not disease specific, and delivered in a group- based format. A special focus should be on the everyday- life of the participants. The most challenging aspect was a present lack of competence and experience among health professionals to moderate self-management support programmes.

**Conclusions:**

The development and design of a relevant and applicable self-management support programme in primary health care should balance the interests of the users with the possibilities and constraints within each municipality. It would be vital to benefit from the closeness of the patients’ every-day life situations. The user informants’ perception of a self-management support programme as a supplement to regular medical treatment represented an expanded understanding of the self-management support concept. An exploring approach should be applied in the development of the health professionals’ competence in practice. The effect of a self-management support programme based on the core characteristics found in this study needs to be evaluated.

## Background

Self-management support has become an important strategy worldwide [1] and plays a central part in chronic disease management [2-6]. Researchers describe it as provision of education and supportive interventions to users from health care professionals and others [7]. Although the existing support programmes [8-10] have a variety of modes, formats, and content [11], features like patient-centeredness and goal-setting skills are commonly included [2,12-14]. Still, the concept of self-management support is ambiguous and its applicability in practice needs to be enhanced [15]. This calls for development of self- management support programmes, especially in primary health care [16]. At the same time, integration of self-management support initiatives in regular health practices is hard to achieve [17]. It represents a challenging task for local authorities and health professionals [7,18,19]. Several studies describe adaptation of an existing programme as an approach often used to develop new support programmes [20-22]. This implies a need to adjust the design to target groups and local norms [23]. Involvement of both users and health care professionals in programme development is highly promoted [16,24], but still seems to be limited in practice. To the best of our knowledge, no studies demonstrate that adaptations and adjustments will be a sufficient approach to support programme development. More studies on this matter should therefore be encouraged. Furthermore, the limited amount of studies found on self-management support programmes in rural and smaller municipalities [25,26] makes it important to investigate which elements should be accounted for in the specific contexts. It may be that different settings create a need for development of different programmes. In a Norwegian context, the White Paper on coordinated health care [27] puts transfer of self-management initiatives from general hospitals to primary health care on the political and public agenda. To address this matter researchers in Central Norway initiate a regional research project on coordinated chronic care. As part of the research project, the present study takes place in the aftermath of a process to develop a specific self-management support programme. Due to a general lack of self-management support programmes in primary health care at the time, the specific programme development allows the researchers to search closer for important factors that may influence the applicability of a support programme in primary health care. The aim of the present study is therefore to explore users’ and health professionals’ perceptions of what would be the vital elements in a self-management support programme applicable in primary health care, how to account for them, and why. This paper describes their perceptions and presents the implications for future programme development and design.

## Method

This was a qualitative interview study among users and primary health professionals. Recruitment and data collection took place in five municipalities in Central Norway in 2010. The Central Norway Regional Committee for Medical and Health Research Ethics approved the study (4.2008.2351). The study was registered at the Norwegian Social Science Data Services (20990).

### Study setting

The Norwegian health system provides general health promotion, disease prevention, curative treatment, rehabilitation, and long- term care services in primary health care [28]. The provisions are mainly public funded and include all inhabitants. Municipalities differ highly according to size, closeness to general hospitals and population density. The municipality populations may range from 215 to 620000 persons [29]. In this study, the populations ranged from 4500 to 182000. The general hospitals provide educational support for patients and their next of kin [30], often with a disease-specific and group-based approach [31].

#### The development of a specific self-management support programme

Prior to the present study, a regional group, with representatives from two user organisations, one regional university hospital, and four municipalities, collaborated with the researchers to develop a specific self-management support programme. A fifth municipality withdrew from the programme development due to a temporary limitation in capacity. A project steering committee made decisions on location in different primary health care practices, target groups of people over 45 years, living with chronic obstructive pulmonary disease (COPD), heart failure, or stroke. In addition, the committee decided the programme structure to be a combination of education and physical exercise. The regional group considered the best mode (group or individual), format (disease - specific or generic), and approach for a support programme to fit into different primary health care practices and run by local health professionals. Relevant topics of content, number of sessions, and total duration of each session were also for them to propose.

All regional group members participated in two joint workshops during a six months period, from March to August 2009 (Table 1). At the initial workshop, presentation of a preliminary programme draft helped to promote reflections and discussions. The draft was based on Lorig’s Chronic Disease Self-Management Program (CDSMP) [12] and medical treatment guidelines [5,6]. In between the joint workshops, two ad-hoc panel groups worked more in-depth on the educational part and the physical exercise part of the programme. Only primary health care professionals attended these panel groups. The process resulted in a more detailed programme draft. Thorough discussions at the second joint workshop led to minor revisions of words and renewed examples in the educational part (Table 2).

**Table 1:** **Illustration of the specific programme development process**

**Programme development**			
**Time**	**Activity**	**Objective**	**Participants**
Mar. 09	Initial workshop	Baseline introduction to the project. Discussions on a preliminary programme draft.	Regional representatives (N = 24);
			user representatives (n = 4)
			hospital units representatives (n = 8)
			primary health care representatives (n = 12)
	Ad-hoc working groups	In-depth considerations on revision needs.	Primary health care professionals (N = 8)
Jun. 09	Second workshop	Joint audit on the revised programme draft. Discuss and agree upon a final programme.	Regional representatives (N = 20);
			user representatives (n = 5)
			hospital units representatives (n = 5)
			primary health care representatives (n = 10)
Aug. 09	Local training sessions	Pre-training on-site.	Group leaders in four municipalities (N = 15)
Sep. 09	Programme application starts in four municipalities

**Table 2:** **Design of a group-based educational and physical training programme (Great) self- management support programme**

**Physical exercise**	**Self-management education**
**0-8 weeks**	
1-hour sessions, twice a week for 8 weeks. Led by local physiotherapists.	2-hour sessions, once a week for 6 weeks. Led by local health professionals.
**Principles:**	**Themes:**
1.Warm-up (15–20 min): exercises for large muscle groups.	1. Establish the group. Assess expectations. Confidentiality.
2. Work-out (30 min); strength, endurance, balance, mobility.	2. Activity and social participation; recommendations and possibilities.
3. Moderate intensity (12–15 on Borg RPE scale) with peaks of higher intensity.	3. Chronic disease(s). Self-management, coping strategies, personal choices.
4. Slow- down, stretching, relaxation (10–15 min).	4. Family, friends, working situations. Chronic disease from relatives’ perspectives.
	5. Communication and cooperation. Interaction with health professionals.
	6. How to move on. Personal goal- setting. Evaluation.
**8 weeks -11 months**	
No supervised exercise activities	3- 4 telephone calls from a local health professional (1 every eight week).
**11-12 months**	
1-hour sessions, once a week for four weeks. Led by local physiotherapists.	One 2 hours follow-up session; Up-date of previous themes, decisions on personal goals after completion at 12 months. Led by local health professionals.

### Recruitment procedures

A purposeful sampling was carried out to involve both users and health professionals as informants. Initially, the main criterion for selection was experience in development or running of a self-management support programme. The lack of relevant support programmes in primary health care made this criterion insufficient in order to ensure response variation. Therefore, also informants with experience from living with a chronic condition (users) or providing regular chronic care in primary care (health professionals) were included (Table 1). The first author contacted all the users and primary health care professionals previously involved in the specific support programme development. She knew them from the previous programme development process. Then, the board leader of a user committee at the university hospital and the administrative leaders in primary health care practices, respectively, recruited the users and health professionals with experience from either living with a chronic disease or providing regular chronic care.

### Informants

The procedures resulted in recruitment of 16 persons (Table 3). Eleven of them were female primary health care professionals; two registered nurses, six physiotherapists, and three occupational therapists. The majority of health professionals were experienced clinicians with more than ten years in service. Four of the five user informants were men. All users were active in user organisations. They got their diagnoses more than five years ago.

**Table 3:** **Distribution of informants**

**Informants**	**Gender**	**Participated in a specific programme development**	**Living with a chronic disease or providing regular treatment**	**Total**
User representatives	4 males	3	2	5
Primary health professionals	All females	7	4	11
In total		10	6	16

### Data collection

Data was collected in a six months period of 2010. Four separate focus group interviews were conducted, three with health professionals and one with user informants. To maintain an intended variation in representation and response the amount of interviews was considered sufficient and satisfactory. Three focus group interviews took place at the university campus. In the most remote municipality, the focus group interview took place at the informants’ working place. They were health professionals with experience from provision of regular chronic care. The length of the focus group interviews varied from 40 to 75 minutes. HSS moderated three of the focus group interviews. MS moderated the first focus group with the health professionals involved in the specific programme development. After a presentation of the purpose of the study, the verbal consents to participate were tape-recorded. Technical problems made it impossible to audiotape the focus group interview with the user informants. Notes were taken. Two of the user informants therefore met with the moderator the next day to safeguard that the notes were correct. This second meeting was audio taped. A semi-structured interview guide was used in each focus group interview [32,33]. This allowed the informants to address the same questions across the groups and still put emphasize on different topics within each group. The main questions asked were; *what is your perception of a self-management support programme in primary health care,* and *what would be the most important elements to account for in a chronic care support programme?* Sub- questions asked explored the contributing factors and/or barriers for a support programme in primary health care and the reasons the informants gave for their perceptions. Both moderators encouraged their informants to discuss freely and ensured that all were heard [32]. Still, MS, who is an experienced moderator, had to put in an extra effort to enhance the dialogue among the informants.

### Data analysis

First author transcribed all the focus group interviews verbatim. Data was analysed by Systematic Text Condensation (STC) [34]. First author distributed the transcripts to the co-authors to secure that at least three authors read each transcript. In line with the STC method, all authors read the transcripts independently to get an overall impression. Then they met to present and discuss their impressions of the material. Based on the discussion all authors agreed on preliminary main themes and perspectives. The first author then coded the interviews according to these themes and wrote a preliminary description of each theme. In an iterative process with meetings where the in- between- meetings work of the first author was discussed, the final categories and sub-themes were decided and agreed upon. Then, to preserve the meanings of the informants, the first author compared the final themes to the original material. Finally, she selected the citations most illustrative.

## Results

The results showed a joint and overall positive expectation to the potential benefits from development of a self-management support programme in primary health care. “It had to come” one informant said. To achieve a more structured service system and enhanced co-operation were vital to all informants and was given as a reason for their positive expectations. Another main result was the joint agreement among the users and the health professionals on core characteristics of a self-management support programme in primary health care. However, the users and the health professionals assigned somewhat different arguments and perspectives to the core characteristics. The characteristics were categorised into three themes: 1) acknowledgement of a generic and group-based approach, 2) a special focus on everyday-life, and 3) the importance of relevant professional competence.

### Acknowledgement of a generic and group-based approach

The informants emphasised that a self-management support programme in primary health care should have a generic, not disease-specific, format and a group-based mode. An argument for this was to ensure better access for the users, closer to their homes. According to the user informants, better access would enhance participants’ possibilities for improvement of their self-management skills. However, factors like municipality size and population density could be barriers for recruitment of a sufficient number of inhabitants with identical diagnoses. Thus, the disease – specific approach would not be a sustainable strategy over time.

*Many municipalities may have few individuals with a specific diagnosis. When health authorities develop self-management programmes (in primary health care) then it is important to meet the needs of most patients, and not just one particular group.* (User informant)

The support for a group-based mode was largely due to the current discrepancies in supply and demands for health professional services. The health professionals viewed a group-based approach less resource demanding than an individual treatment approach. Thereby, their workload would probably not increase any further. Moreover, the user informants argued that the group-based approach would improve social peer-support. They expected that improved social support could have a positive impact on participants’ well-being and subsequently on their engagement in self-management.

*It will reduce the workload. Groups are time saving. It is a lack of resources anyway. We reach more people working with groups* (Health professional)

*This is not about waffles and coffee, but about the possibility to become a part of a fellowship. It has something to do with psychological aspects* (User informant).

All informants expected a generic and group-based format to increase the disparity of composition between and within groups. This indicated a need to account for individual and local adjustments. Especially the user informants viewed such adjustments as vital to enhance the user orientation of support programmes, and make them more relevant to participants. However, the provision of individualised support within a generic and group-based format represented a major challenge to the health professionals.

### A special focus on everyday-life

The user informants highlighted the need to include an everyday-life perspective in support programmes in primary health care. An argument put forward was that having an everyday- life perspective would increase the relevance for the participants. Unless hospitalised, the users opted to limit their focus on medical diagnoses and disease-specific topics. Especially in stable disease periods, it was essential for them to maintain a focus on well-being, coping and everyday-life functioning. Thus, they argued that self-management support programmes in primary health care should be a supplement to regular medical care and individual encounters with the physicians. In particular, more focus should be on aspects of co-morbidities, users’ psychological and social situations.

*What diagnosis they may have is not of major interest. If they have this or that diagnosis, they all have in common a reduction in their level of activity. This* [programme] *is an addition* (User informant).

None of the user informants expected the health professionals to possess personal experience from living with a chronic disease. Thus, they promoted inclusion of experienced user representatives as partners in planning and running of a support programme. None of the health professionals mentioned this option. Still, the health professionals were not confident in how to account properly for the everyday-life experiences and needs of the participants. Consequently, one of them highlighted that the combination of physical exercises and self-management educational sessions would reduce the perceived challenge to some extent. She also viewed the programme structure to be favourable to account for different learning styles. User representatives accentuated the possible implications of the programme structure to the participants’ perception of safety. The emphasis on structured sessions and everyday-life would imply more feedback and follow-ups after practicing skills on their own. All the informants expected that the combination would enhance and increase the participants’ motivation to further personal commitment into self-management work.

*I do not expect health professionals to understand how it is to have a chronic disease. Still, they need to know something about psychological aspects, which concern all diagnoses. They may listen to the participants and try to imagine how difficult it is for anybody* (User informant).

*A dedicated time for information and education, and another one to test it in practice, that provides for new learning and processes for the participants.* (Health professional)

### The importance of relevant professional competence

All informants considered the competence of group leaders as most important to secure the quality of the programme provision in practice. They expected the competence to be relevant and up-dated. However, neither the health professionals themselves nor the user informants were confident if there would be a sufficient amount of professionals to run a self-management support programme, especially in smaller municipalities.

*I wonder if small municipalities can manage to make this new service sustainable. They lack both the competence and the capacity* (User informant).

The health professionals described discrepancies related to their current competence and the one needed to run the educational part of a group-based programme. They questioned if they possessed enough updated medical knowledge, and expressed a lack of competence in facilitating group processes. Moreover, they expected a need to apply their current competence in a different way.

*This arrangement with communication in a group, it differs from other advice and instructions. This is a new way of working. It is a new focus and commitment towards the dialog and processes in the group* (Health professional).

The user informants and health professionals suggested different options to achieve and develop the relevant competence needed. Health professionals preferred to achieve most of the competence through experience. This meant that health professionals just had to start running the support programme, explore pitfalls and success, and gain more knowledge and skills along the road. Although they felt challenged by this approach, they expected to be able to cope with it. They expressed familiarity with this way of working in other professional situations.

*It is exciting to be part of something new. I think we just have to get started! Although I expect challenges ahead, gradually I will be more experienced* (Health professional).

User informants acknowledged the challenges expressed by the health professionals, but they did not fully support their exploratory approach. According to one of the user informants, selection of the “right persons” to run the programme would be a better way to overcome the challenges. Right persons meant health professionals with empathy and good communication skills. According to him, it would be important to enhance the health professionals’ educational qualifications before they ought to run a self-management programme.

## Discussion

In the present study focus was on users’ and health professionals’ perceptions of a self-management support programme applicable in primary health care and the implications for future programme development and design. For the purpose of the study, three topics will be discussed; 1) self-management support as a supplement to medical treatment, 2) a relevant and challenging design, and 3) professional competence development.

### Self-management support as a supplement to medical treatment

In this study, the user informants viewed self-management support as a supplement to medical treatment. The result could be interpreted as a call for the present medical care to correspond better with the users’ needs and interests [35]. This would imply inclusion of more group-based provisions with a broader focus on the participants’ psychosocial situation, coping, and well-being in stable periods of their lives. A broader perception of self-management support corresponded well to the study context and the political decisions on reforms in integrated care in Norway. Moreover, it could mean an expansion of the self-management support concept. To the best of our knowledge, this aspect has not been highlighted in previous studies, at least not from the perspective of the users. According to Kawi [15] how people think about self- management support would have an impact on what should be included in the support concept. It would also provide guidance for implementation and practice outcomes. A broader understanding of self-management support, as a supplement to medical treatment, reflected a need to put emphasis on what Crespo [36] called “getting a system into place”. The users interpreted a structured system to represent an assurance for a sustainable provision. Yet, it would be essential to avoid that the supplemental role of a self-management support programme turns into just another task for the health providers. Then the demands on personnel resources would probably increase and the sustainability of a support programme in primary health care would still be vulnerable. Therefore, to achieve positive outcomes, integration of a new and supplemental support programme into the regular health system should be enhanced [36]. According to Renders [37] dedicated roles for team members would be one strategy to enhance the health providers’ awareness of self- management support.

### A relevant and challenging design

Lorig has previously successfully introduced the generic and group-based approach in programme design found in this study [12,38]. In the present study, the generic approach was an appealing option to reach more people within the constraints of time and personnel [16]. Still, the active user role inherent in self-management support would disfavour persons who are unable to hold such a position [39]. Although the group format was associated with enhanced social support for individuals [40], the joint preference for this format was mainly related to demographic and geographic conditions. This reflected a need to balance between user needs and possibilities within the actual setting in the development of an applicable self-management support programme. Another key feature in the design of the programme was the special focus on everyday-life. In primary health care, the strength of the programme would be its closeness to the local situations and contexts, where people practice self-management and live for most of the time. To make use of this advantage would improve the possibilities to understand the participants’ experiences in their ordinary environments [41]. Hence, it was a surprise, at least to the researchers, to discover that only the user informants pinpointed the need for a closer collaboration between user organisations and primary health care. Collaboration in planning and running of support initiatives has been formalised in the support initiatives at the general hospitals for a long time. It should therefore be an easy task to consider and incorporate the collaborative approach in a support programme in primary health care as well. It might be that the health professionals merely were unfamiliar to the idea of involving user representatives in programme running. Still, an enhanced collaboration should have the potential to reduce the local challenges caused by lack of personnel resources.

### Professional competence development

Similar to Lawn and Schoo [11] informants in this study viewed the lack of competence in running a self- management support programme among primary health care professionals to be a major challenge in development of an applicable programme. Since relevant and available competence is imperative to gain positive effects from implementation [42], discrepancies put strains especially on health care and its providers to bridge the gap. Our results were similar to studies on self-management support that emphasized inter-professional cooperation, [11,43] and strategies to improve user-involvement [44] as core skills for health professionals engaged in self-management support. In line with Liaw and colleagues [45], knowledge and skills in adjustments to cultural and social norms would be important to enhance in support programme development and running. In the Norwegian White Paper on coordination [27], transfer of relevant knowledge from hospitals to primary health care was mentioned as a prerequisite. If transfer implied disease-specific, medical, knowledge, our results did not correspond entirely to that perception. The results pinpointed the need to improve the health professionals’ communication skills and their roles as moderators in processes of change for individuals within a group. Therefore, strengthening the health pedagogic competence would be more adequate and vital to enhance. Development of professional competence should include theoretical knowledge, practical skills, and personal competence [46]. Despite its relevance, neither transfer of medical knowledge across service levels, nor adjustments according to the specific local settings would probably be sufficient approaches to improve and develop adequate professional competence. To deal with questions or challenges that lack straightforward evidences or answers, the health professionals fronted a need to build competence through real-world situations. This action oriented and explorative approach could be an adequate strategy to promote a necessary sense of ownership to change processes and outcomes [47] and should be addressed more. Moreover, to build competence, achieve changes and quality improvements, it would be necessary to organise and perform a structured process of continual reflections and evaluations [48].

### Strengths and limitations

A major strength of this study was that it took into account the perspectives of both user representatives and health professionals. The joint agreement between user representatives and health professionals on core characteristics, despite different perspectives, may have increased the validity of the results. However, one limitation was that the study took place in Norway. The majority of municipalities have low population density apart from some larger cities. The inclusion of a city in this study alleviated this limitation somewhat. Still, generalisation of the findings to more densely populated countries would require careful considerations. This would also be true for countries without a strong foundation for service provision in primary health care. The lack of general practitioners among the informants may have contributed to the limited focus on medical diagnosis. The first author was actively involved in the programme development process prior to the present study. In order to reduce bias on the results, the researchers put a special attention on this matter throughout the entire data collection and analysis. One example would be that MS moderated the interview with those informants who had been involved in the programme development process.

## Conclusions

The findings of this study implied that development and design of a relevant and applicable self-management support programme in primary health care should balance the interests of the users with the possibilities and constraints within each municipality. It would be vital to benefit from the closeness of the patients’ every-day life situations, and to take into consideration constraints like few patients with identical diagnosis, and few employees with relevant knowledge and competence in running a self-management support programme. The development of the health professionals’ competence in practice needs to comprise an exploring approach to improve inter-professional cooperation, communication skills, process facilitation, and support management in groups. From the user perspective, the perception of a self-management support programme as a supplement to regular medical treatment represented an expanded understanding of the self-management support concept. The effect of a self-management support programme based on the core characteristics found in this study needs to be evaluated.

## Abbreviations

COPD: Chronic obstructive pulmonary disease
CDSMP: Chronic disease self- management program
STC: Systematic text condensation
